# Elusive endocarditis: a diagnosis by physical examination

**DOI:** 10.1093/ehjcr/ytaf169

**Published:** 2025-04-07

**Authors:** Stephanie A Saey, David M Harmon, Michael W Cullen

**Affiliations:** Department of Internal Medicine, Mayo Clinic, 200 1st St SW, Rochester, MN 55905, USA; Department of Cardiovascular Medicine, Mayo Clinic, 200 1st St SW, Rochester, MN 55905, USA; Department of Internal Medicine, Mayo Clinic, 200 1st St SW, Rochester, MN 55905, USA; Department of Cardiovascular Medicine, Mayo Clinic, 200 1st St SW, Rochester, MN 55905, USA

A 76-year-old male with a history of right lung adenocarcinoma and prior lobectomy presented to cardiology for newly diagnosed atrial fibrillation and mitral valve regurgitation. He reported ongoing dyspnoea, fatigue, lower extremity oedema, and a recent episode of malaise and chills. A physical exam demonstrated a low-grade holosystolic murmur at the apex, a miniscule, longitudinal red-brown haemorrhage under the left middle fingernail (*Figure 1A*), and an erythematous papule on the left middle finger (*Figure 1B*). Given the dermatologic abnormalities and possible infectious symptoms, two sets of peripheral blood cultures were obtained and grew *Streptococcus mitis* in 4/6 bottles within 16 h.

**Figure 1:**
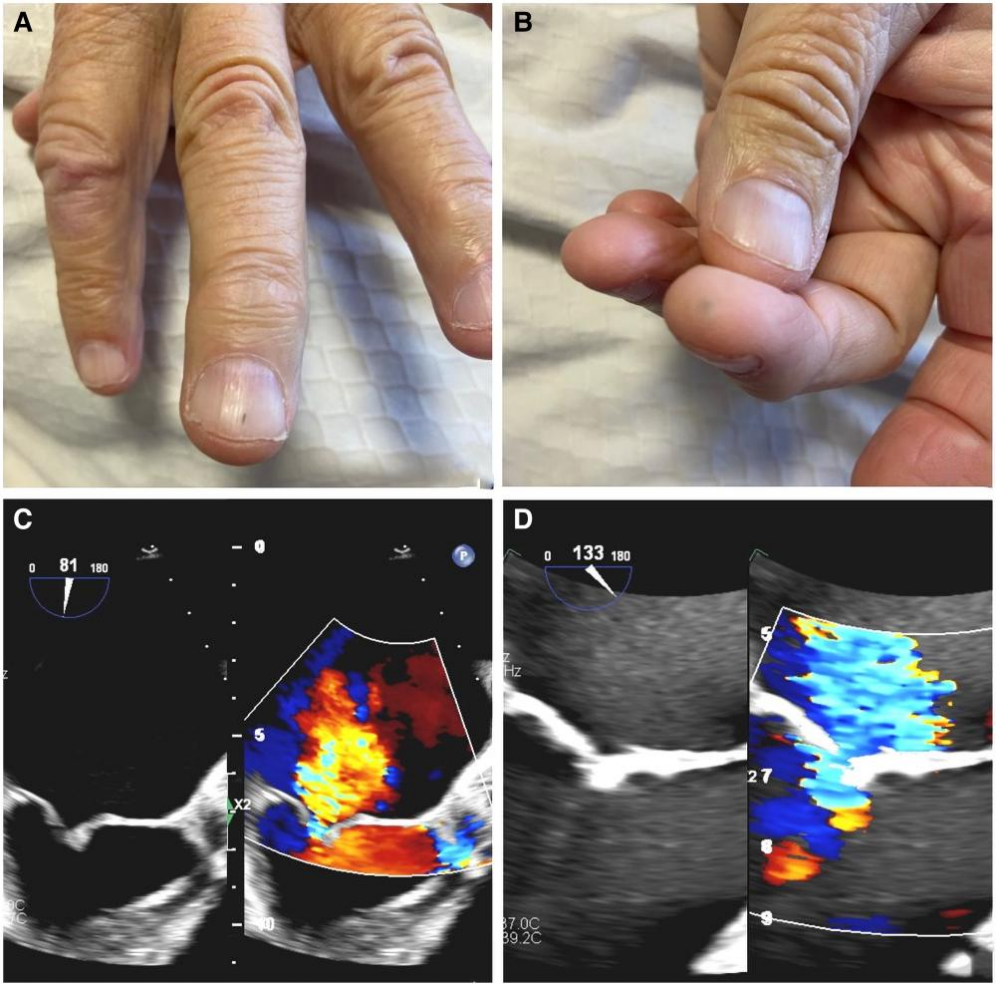
Clinical and echocardiographic findings in a patient with infective endocarditis. (*A*) Splinter haemorrhage and (*B*) Janeway lesion observed during the initial clinical presentation. (*C*) Initial transoesophageal echocardiography demonstrating thickened mitral valve leaflet tips (5.5 mm) with known moderate mitral regurgitation. (*D*) Follow-up transoesophageal echocardiogram showing no significant change in leaflet thickness or degree of mitral regurgitation. Nyquist limit was adjusted for MR quantification; image selection was based on availability and optimal visualisation of the MR jet.

The patient was hospitalized, and a transesophageal echocardiogram (TEE) showed a thickened mitral valve with known moderate regurgitation but no vegetations (*Figure 1C*). All repeat blood cultures were negative, and a second TEE 2 weeks later showed no significant changes or evidence of infection (*Figure 1D*). This patient met the 2023 European Society of Cardiology (ESC) diagnostic criteria for possible infective endocarditis (IE) with one major (high-risk pathogen; *Streptococcus mitis*) and one minor criterion (Janeway lesion). Despite two negative TEEs, he initiated treatment for presumed subclinical IE.^1^ At follow-up, he noted marked improvement in fatigue and dyspnoea with no further infectious symptoms.

Microembolic phenomena, including Janeway lesions and splinter haemorrhages, occur in ∼5%–10% of IE cases.^2^ Janeway lesions are non-tender, erythematous macules and papules on the palms and hypothesized to result from septic emboli. They represent a minor criterion in the 2023 ESC criteria for IE, while splinter haemorrhages lack specificity and are not included as a formal diagnostic criterion. Clinicians can easily miss these examination findings in the outpatient setting when suspicion for ongoing infection is low. As this case demonstrates, these findings may change management, underscoring the critical importance of physical examination in the early diagnosis of IE.

**Consent:** Written informed consent for submission and publication was obtained from the patient in accordance with publication ethics set out by the Committee on Publication Ethics.

**Funding:** None declared.

## Data Availability

All data underlying this article are available in the article.
